# Assessment of the Efficiency of Tulsi Extract as a Locally Administered Medication Agent and Its Comparison With Curcumin in the Treatment of Periodontal Pockets

**DOI:** 10.7759/cureus.54619

**Published:** 2024-02-21

**Authors:** Ankita Agrawal, Anant Ragav Sharma, Varsha Rathod, Anand Bhatnagar, Pallavi Amol Khale, Priyanka Tidke, Dhaval Mehta, Debojyoti Mazumder

**Affiliations:** 1 Department of Conservative Dentistry and Endodontics, Buddha Institute of Dental Sciences and Hospital, Patna, IND; 2 Department of Periodontics, Pacific Dental College and Hospital, Udaipur, IND; 3 Department of Periodontology, Dr. D. Y. (Dnyandeo Yashwantrao) Patil School of Dentistry, Navi Mumbai, IND; 4 Department of Periodontics, Jaipur Dental College, Jaipur, IND; 5 Department of Dentistry, Rajiv Gandhi Medical College and Chhatrapati Shivaji Maharaj Hospital, Thane, IND; 6 Department of Oral Medicine and Radiology, MGM (Mahatma Gandhi Mission) Dental College and Hospital, Navi Mumbai, IND; 7 Department of Oral Medicine and Radiology, Narsinbhai Patel Dental College and Hospital, Sankalchand Patel University, Visnagar, IND; 8 Department of Conservative Dentistry and Endodontics, Kusum Devi Sunderlal Dugar Jain Dental College and Hospital, Kolkata, IND

**Keywords:** scaling, locally administered medication, periodontal pocket, curcumin, tulsi

## Abstract

Introduction: The use of locally administered medication (LAM) agents such as minocycline, metronidazole, and tetracycline as antimicrobials has drawbacks, including the development of microorganism resistance, exorbitant pricing, and limited accessibility. Thus, there is a need for safer and more affordable alternatives. Numerous natural therapies have been found to be superior in this situation. In this study, the efficacy of tulsi extract as a LAM agent was assessed and it was compared with curcumin, which is currently used for the treatment of periodontal pockets.

Methods and materials: There were three categories: each category had 30 sites. Category 1 sites underwent scaling along with root planing (SRP) solely, Category 2 sites received curcumin extract as LAM in the periodontal pocket in addition to SRP, and Category 3 sites received tulsi extract as LAM in the periodontal pocket in addition to SRP. The stent was used to ensure consistent and unbiased measurements on the 30th day after treatment. Clinical attachment level (CAL) and probing pocket depth (PPD) were measured at six points around each tooth.

Results: The reduction in values of periodontal parameters such as BAPNA (Nα-benzoyl-DL-arginine-p-nitroanilide) assays, modified sulcus bleeding index (mSBI), gingival index (GI), plaque index (PI), CAL, and PPD in sites within Category 1, Category 2, and Category 3 was statistically significant. The decrease in BAPNA assay results indicates that tulsi extract is more effective than curcumin gel at eradicating red-complex bacteria. Although not significantly different, the decrease in PI and GI was observed to be greater when curcumin jelly was used. This suggests that curcumin jelly has a stronger impact on reducing plaque, which in turn decreases gingival inflammation.

Conclusion: Based on the overall results of the study, it can be said that both tulsi and curcumin have similar effectiveness in reducing periodontal markers.

## Introduction

There is a need for safer and more affordable alternatives to the use of antimicrobials like minocycline, metronidazole, and tetracycline as locally administered medication (LAM) agents. This is because these antimicrobials have drawbacks such as the emergence of microorganism resistance, exorbitant pricing, and limited accessibility [1,2]. It has been discovered that several natural remedies are superior options in this case. When administered alone or in conjunction with other types of antibiotics, curcumin produced from turmeric (*Curcuma longa*) has demonstrated its anti-inflammatory and antibacterial effects [3-6]. When administered as an external application, lozenges and LAM ingredients are introduced into the periodontal pocket. Additionally, when used as a mouth rinse, they are useful in the management of periodontitis [7,8].

The volatile oils, including curcumin, present in *C. longa* are known to have anti-inflammatory properties. Oral curcumin was discovered to be equally effective in treating acute inflammation as cortisone compounds or phenylbutazone compounds in treating chronic inflammation [9]. When administered orally to the control group, *C. longa* significantly decreased the inflammatory swelling caused by Freund's adjuvant-induced arthritis in rats [6]. Curcumin was found to reduce neutrophil aggregation, which is a marker of inflammation [5]. *C. longa*, a plant with anti-inflammatory properties, can inhibit neutrophil activity and the generation of prostaglandins from arachidonic acid in inflammatory conditions [4]. Curcumin can also be applied topically to reduce the signs and symptoms of allergic reactions and reactive skin disorders [10]. At relatively low doses of 10-15 g/ml, curcumin almost entirely suppresses the proliferation of *Treponema denticola*, *Fusobacterium nucleatum*, *Prevotella intermedia,* and *Porphyromonas gingivalis.* It also has an antimicrobial effect on pathogenic bacteria associated with periodontal disease [10]. It is more suitable as a topical medicine rather than for oral consumption due to its plasma half-life of approximately 6.77 hours after ingesting 10-12 mg of curcumin orally [11].

*Ocimum sanctum* (holy basil or tulsi), another herb that is frequently found in mouth rinses, is used for the management of periodontitis [11] due to its antibacterial, immunoregulatory, wound-healing, and anti-inflammatory effects. Eugenol and methyl eugenol are the two essential oils found in tulsi. It has been suggested that Tulsi possesses immunomodulatory properties, potentially bolstering the host's response to infections by elevating levels of interferon, interleukin-4, and T helper cells. This mechanism implies a potential enhancement in the body's ability to combat pathogens through the regulation of immune factors [11]. However, tulsi has not yet been studied to determine its efficacy as a LAM agent for treating periodontal pockets. Therefore, this study aims to evaluate the effectiveness of tulsi extract as a LAM agent and compare it to curcumin, which is currently being used to manage periodontal pockets.

## Materials and methods

This clinical study was conducted at the Department of Periodontics, Buddha Institute of Dental Sciences and Hospital, Patna, India,for two months from June 2021 to August 2021. The study was approved by the Institutional Ethics Committee of Buddha Institute of Dental Sciences and Hospital (approval number: IEC/BIDSH/2021/345A). A total of 30 patients were enrolled and informed consent was taken from them. Inclusion criteria were systemically healthy subjects between the ages of 35 and 55 years, with pocket depths ranging from 5 mm to 8 mm, and a minimum of 20 teeth that were intact. Three distant locations in different mouth quadrants were included. The study excluded patients who were undergoing systemic antibiotic therapy, using antibacterial mouth rinses, receiving orthodontic treatment, wearing prosthetic teeth, nursing or pregnant, or had an allergy to any of the medications used in the investigation.

To ensure homogeneity among study locations, the first molars of the mandibular arch and maxillary arch were used. In this study, a total of 90 regions were chosen. There were three categories; each category had 30 research locations. Category 1 sites underwent scaling along with root planing (SRP) solely, Category 2 sites received curcumin extract as LAM in the periodontal pocket in addition to SRP, and Category 3 sites received Tulsi extract as LAM in the periodontal pocket in addition to SRP.

Study phases

When used as a LAM agent, the dosage of tulsi extract's antibacterial activity is unknown. Consequently, the investigation was conducted in two stages. In stage one, the minimum inhibitory dosage of tulsi concentration was calculated for its antibacterial effect. The second phase was designed to use and compare curcumin cream (Curenext gel®) and an extract from tulsi as LAM medications for managing periodontal pockets. Curenext gel was used as it contains curcumin in its optimal concentration as of now.

The Initial Phase

With the aid of freshly distilled water in a gel foundation, various tulsi extract concentrations (2%, 4%, 6%, 8%, and 10%) were prepared and evaluated using the time-kill curve approach. The evaluation was carried out against pathogens commonly associated with periodontal diseases, such as *Tannerella forsythia*, *Porphyromonas gingivalis,* and *Aggregatibacter actinomycetemcomitans *[12,13]. The findings demonstrated that when tulsi extract concentrations approached 10%, there was a significant antibacterial effect. Therefore, in the present investigation, 10% tulsi extract was administered as an LAM agent.

The Second Phase

An acrylic stent was used to ensure consistent and unbiased measurements on the 30th day after treatment; it had a notch to insert the UNC 15 probe (Hu-Friedy Mfg. Co., LLC, Illinois, United States). Clinical attachment level (CAL) and probing pocket depth (PPD) were measured at six points around each tooth. There were recordings of gingival index (GI), modified sulcus bleeding index (mSBI), and plaque index (PI) according to Loe and Silness (1963), Mombelli et al., and Loe and Silness (1964), respectively [14-16].

Using sanitized Gracey curettes (Hu-Friedy Mfg. Co., LLC), specimens of subgingival plaque were obtained [17] and transferred in reduced transportation solvent for N-benzoyl-L-arginine-p-nitroanilide (BAPNA) testing. By assessing the expression of trypsin-like enzymes in red complex periodontal bacteria, the BAPNA analysis was applied to examine the impact of LAM agents on decreasing the quantity of the red complex bacteria. The BAPNA test is capable of rapidly measuring and quantifying the activity of red complex periodontal bacteria that exist in plaque specimens. This is done by calculating the amount of trypsin-like enzyme in terms of nanomoles of the substance per 60 seconds per milligram of wet weight of dental plaque [18,19].

Without prior flushing with an antimicrobial mouth rinse, a complete single-session SRP was performed after collecting plaque specimens. Category 1 sites received only SRP treatment, Category 2 sites received SRP followed by Curenext gel (curcumin), and Category 3 sites received SRP followed by tulsi extract. Following the procedure, the pocket was sealed with periodontal packing. Participants were instructed to brush using a modified Bass approach and to refrain from using mouth rinse or any other medications for the duration of the study. On the 30th day after the procedure, each site underwent further evaluation for clinical and microbiological data.

Statistical evaluation

The data were analyzed using the trial version of SPSS Statistics for Windows, Version 17.0 (Released 2008; SPSS Inc., Chicago, United States). Calculations were made to determine the prevalence of the result variable and its 95% CIs. A Wilcoxon signed-rank test (as parametric test) was conducted assuming that the results of the BAPNA assays, mSBI, GI, PI, CAL, and PPD followed a normal distribution and that the data for all three categories were also normally distributed. Data were compared within categories from baseline to day 30 post procedure using the unpaired t-test and between categories from baseline to day 30 post procedure using the paired t-test.

## Results

The mean value of PPD in Category 1 at baseline was 5.23±0.57, while it was 4.41±0.72 on the 30th day of follow-up. The reduction in PPD was statistically significant (p<0.001). The mean value of PPD in Category 2 at baseline was 5.40±0.67, while it was 4.39±0.84 on the 30th day of follow-up. The difference was statistically significant (p<0.001). On comparing the values of PPD at baseline (p=0.23) and 30-day follow-up (p=1.01) between sites in categories 1 and 2, there was no statistically significant difference. The mean value of CAL in category one at baseline was 4.86±0.43, while it was 4.21±0.68 on the 30th day of follow-up. The change in CAL was statistically significant (p<0.001). The mean value of CAL in Category 2 at baseline was 4.99±0.84, while it was 4.17±0.79 on the 30th day of follow-up. The difference was statistically significant (p<0.001). On comparing the values of CAL at baseline and 30-day follow-up between sites in categories 1 and 2, there was no statistically significant difference. 

The mean value of PI in Category 1 at baseline was 2.36±0.52, while it was 1.12±0.41 on the 30th day of follow-up. The change in PI was statistically significant (p<0.001). The mean value of PI in Category 2 at baseline was 2.26±0.63, while it was 0.79±0.42 on the 30th day of follow-up. The difference was statistically significant (p<0.001). On comparing the values of PI at baseline and 30-day follow-up between sites in categories 1 and 2, there was no statistically significant difference. The mean value of GI in Category 1 at baseline was 1.77±0.37, while it was 0.81±0.36 on the 30th day of follow-up. The change in GI was statistically significant (p<0.001). The mean value of PI in Category 2 at baseline was 1.69±0.30, while it was 0.76±0.30 on the 30th day of follow-up. The difference was statistically significant (p<0.001). On comparing the values of GI at baseline and 30-day follow-up between sites in categories 1 and 2, there was no statistically significant difference. 

The mean value of mSBI in Category 1 at baseline was 1.91±0.52, while it was 1.24±0.85 on the 30th day of follow-up. The change in mSBI was statistically significant (p<0.001). The mean value of mSBI in Category 2 at baseline was 1.84±0.56, while it was 0.84±0.60 on the 30th day of follow-up. The difference was statistically significant (p<0.001). On comparing the values of mSBI at baseline and 30-day follow-up between sites in categories 1 and 2, there was no statistically significant difference. The mean value of BAPNA in Category 1 at baseline was 3.65±1.32, while it was 1.63±1.01 on the 30th day of follow-up. The change in BAPNA was statistically significant (p<0.001). The mean value of BAPNA in Category 2 at baseline was 3.65±1.70, while it was 1.12±1.18 on the 30th day of follow-up. The difference was statistically significant (p<0.001). On comparing the values of mSBI at baseline and 30-day follow-up between sites in categories 1 and 2, there was no statistically significant difference (Table 1).

**Table 1:** Comparison of periodontal parameters in study participants of Category 1 and Category 2 at baseline and 30th day post procedure PPD: Probing pocket depth; CAL: Clinical attachment level; PI: Periodontal index; GI: Gingival index; mSBI: Modified sulcus bleeding index; BAPNA: N-benzoyl-L-arginine-p-nitroanilide Values given as mean±SD

Variables	PPD	CAL	PI	GI	mSBI	BAPNA
Baseline						
Category 1	5.23±0.57	4.86±0.43	2.36±0.52	1.77±0.37	1.91±0.52	3.65±1.32
Category 2	5.40±0.67	4.99 ±0.84	2.26±0.63	1.69±0.30	1.84±0.56	3.65±1.70
P value	0.23	0.63	0.67	0.52	0.78	1.10
30^th^ day						
Category 1	4.41±0.72	4.21 ±0.68	1.12±0.41	0.81 ±0.36	1.24±0.85	1.63± 1.01
Category 2	4.39 ±0.84	4.17 ±0.79	0.79 ±0.42	0.76±0.30	0.84±0.60	1.12±1.18
P value	1.01	0.96	0.005	0.69	0.22	0.29

The mean value of PPD in Category 3 at baseline was 5.26±0.52, while it was 4.22±0.69 on the 30th day of follow-up. The difference was statistically significant (p<0.001). On comparing the values of PPD at baseline and 30-day follow-up between sites in category one and category three, there was no statistically significant difference. The mean value of CAL in Category 3 at baseline was 4.90±0.52, while it was 3.92±0.44 on the 30th day of follow-up. The difference was statistically significant (p<0.001). On comparing the values of CAL at baseline and 30-day follow-up between sites in categories 1 and 3, there was no statistically significant difference. 

The mean value of PI in Category 3 at baseline was 1.97±0.61, while it was 0.94±0.51 on the 30th day of follow-up. The difference was statistically significant (p<0.001). On comparing the values of PI at baseline and 30-day follow-up between sites in categories 1 and 3, there was no statistically significant difference. The mean value of GI in Category 3 at baseline was 1.72 ±0.37, while it was 0.82±0.42 on the 30th day of follow-up. The difference was statistically significant (p< 0.001). On comparing the values of GI at baseline and 30-day follow-up between sites in categories 1 and 3, there was no statistically significant difference. The mean values of mSBI in Category 3 at baseline was 1.97±0.46, while it was 0.77±0.52 on the 30th day of follow-up. The difference was statistically significant (p<0.001). On comparing the values of mSBI at baseline and 30-day follow-up between sites in categories 1 and 3, there was no statistically significant difference. 

The mean value of BAPNA in Category 3 at baseline was 3.61±1.53, while it was 0.57±0.52 on the 30th day of follow-up. The change in BAPNA was statistically significant (p<0.001). On comparing the values of mSBI at baseline and 30-day follow-up between sites in categories 1 and 3, there was statistically significant difference (p=0.001). It showed that SRP with tulsi was found to reduce the quantity of red complex bacteria more than SRP alone (Table 2).

**Table 2:** Comparison of periodontal parameters in study participants of Category 1 and Category 3 at baseline and 30th day post procedure PPD: Probing pocket depth; CAL: Clinical attachment level; PI: Periodontal index; GI: Gingival index; mSBI: Modified sulcus bleeding index; BAPNA: N-benzoyl-L-arginine-p-nitroanilide Values given as mean±SD

Variables	PPD	CAL	PI	GI	mSBI	BAPNA
Baseline						
Category 1	5.23 ±0.57	4.86 ±0.43	2.36±0.52	1.77±0.37	1.91 ±0.52	3.65 ±1.32
Category 3	5.26 ±0.52	4.90 ±0.52	1.97 ±0.61	1.72 ±0.37	1.97 ±0.46	3.61±1.53
P value	0.99	0.88	0.21	0.74	0.20	1.04
30^th^ day						
Category 1	4.41 ±0.72	4.10±0.57	1.12 ±0.41	0.81 ±0.36	1.24±0.85	1.63±0.11
Category 3	4.22 ±0.69	3.92±0.44	0.94 ±0.51	0.82±0.42	0.77 ±0.52	0.57 ±0.52
P value	0.20	0.34	0.28	0.98	0.08	0.001

On comparing the values of PPD, CAL, PI, GI, mSBI, and BAPNA at baseline and 30-day follow-up between sites in categories 2 and 3, there was no statistically significant difference. However, the values were lower in SRP with curcumin as compared to SRP with tulsi (Table 3). 

**Table 3:** Comparison of periodontal parameters in study participants of category 2 and category 3 at baseline and 30th day post intervention PPD: Probing pocket depth; CAL: Clinical attachment level; PI: Periodontal index; GI: Gingival index; mSBI: Modified sulcus bleeding index; BAPNA: N-benzoyl-L-arginine-p-nitroanilide Values given as mean±SD

Variable	PPD	CAL	PI	GI	mSBI	BAPNA
Baseline						
Category 2	5.40 ±0.67	4.99±0.84	2.26±0.63	1.69 ±0.30	1.84±0.56	3.65±1.70
Category 3	5.26 ±0.52	4.81 ±0.52	2.05 ±0.61	1.72 ±0.37	1.97 ±0.46	3.61±1.53
P value	0.29	0.77	0.44	0.85	0.48	1.05
30^th^ day						
Category 2	4.28±0.73	4.06±0.68	0.68±0.31	0.76±0.31	0.84±0.60	1.12±1.18
Category 3	4.01±0.73	3.92±0.44	0.83±0.40	0.82±0.42	0.77±0.72	0.57±0.52
P value	0.28	0.52	0.27	0.63	0.87	0.08

It was found that there was an improvement in parameters like PPD, CAL, PI, GI, mSBI, and BAPNA in all three categories on intra-category comparison.

## Discussion

The use of antimicrobials, such as minocycline, metronidazole, and tetracycline, as LAM agents, have drawbacks due to the development of microorganism resistance, exorbitant pricing, and inaccessibility [20,21]. Thus, there is a need for safer and more affordable alternatives. Numerous natural therapies have been found to be superior in this situation. In this study, the efficacy of tulsi extract as a LAM agent was assessed and compared with curcumin, which is currently used for the treatment of periodontal pockets.

The reduction in values of periodontal parameters such as BAPNA assays, mSBI, GI, PI, CAL, and PPD in sites in Category 1 was statistically significant in the current study. These findings were similar to the results of research carried out by Cugini et al, Yang et al, and Dhalla et al. [21-23]. They also indicated that SRP is effective in reducing parameters associated with periodontal disease on its own.

Curcumin derived from turmeric (*C. longa*) has been proven to have anti-inflammatory and antibacterial effects when used alone or in combination with other antibiotics [24,25]. This treatment regimen demonstrates efficacy in combating periodontitis through multiple modalities, including topical application, lozenges infused with LAM for direct application to periodontal pockets, and a complementary mouth rinse [8]. The volatile oils in *C. longa*, which contain curcumin, are recognized for their anti-inflammatory properties. It has been shown that oral curcumin is just as effective at treating acute inflammation as cortisone or phenylbutazone at treating chronic inflammation [20,24]. When taken orally, as opposed to the control group, *C. longa* significantly decreased the inflammatory swelling caused by Freund's adjuvant-induced arthritic conditions in rats.

Curcumin was found to reduce neutrophil aggregation, which is a marker of inflammation [5]. In inflammatory conditions, *C. longa*, which possesses anti-inflammatory properties, can suppress neutrophil activity and prostaglandin production from arachidonic acid. To alleviate the signs and symptoms of allergic reactions and reactive skin conditions, curcumin can also be applied topically [25,26]. Curcumin has an antibiotic effect on pathogenic bacteria linked to periodontal disease and effectively inhibits the growth of *T. denticola*, *F. nucleatum*, *P. intermedia, *and *P. gingivalis* at relatively low dosages of 10-15 g/ml [20]. Due to its plasma half-life of approximately 6.77 hours following oral administration of 10-12 mg of curcumin, it is better suited as a topical medication rather than for oral consumption [20,24].

The reduction in values of periodontal parameters such as BAPNA assays, mSBI, GI, PI, CAL, and PPD in sites in Category 2 was statistically significant in the current study. These findings were similar to the results of studies carried out by Hugar et al., Varghese et al., Anitha et al., and Gottumukkala et al. [20,24-26]. They found that curcumin with SRP is effective in reducing parameters associated with periodontal disease.

Another plant that is frequently included in mouth rinses is *O. sanctum* (tulsi), which is used for its antibacterial, immunoregulatory, wound-healing, and anti-inflammatory properties in the treatment of periodontitis [27]. Both methyl eugenol and eugenol are present as essential oils in tulsi. The effectiveness of tulsi as a LAM agent for treating periodontal pockets has not yet been investigated [28]. The reduction in values of periodontal parameters such as BAPNA assays, mSBI, GI, PI, CAL, and PPD in sites Category 3 was statistically significant in the current study. These findings were similar to the results of research carried out by Gupta et al. and Hosamane et al. [27,28]. They discovered that tulsi with SRP is effective in reducing parameters associated with periodontal disease.

The decrease in BAPNA assay results indicates that tulsi extract is more effective than curcumin gel at eradicating red complex bacteria. Although not significantly different, the decrease in PI and GI was observed to be greater when curcumin jelly was used. This suggests that curcumin jelly has a stronger impact on reducing plaque, which in turn decreases gingival inflammation. We can, therefore, conclude from the current study results that both herbs have comparable efficacy in reducing periodontal markers. On comparing the values of BAPNA assays, mSBI, GI, PI, CAL, and PPD at baseline and 30-day follow-up between sites of Category 1 and Category 2, there was a better response in terms of periodontal parameters in Category 2. However, there was no statistically significant difference. Upon comparison of BAPNA assay values, mSBI, GI, PI, CAL, and PPD between Category 1 sites and the other two categories, a more favorable response in terms of periodontal parameters was observed in Category 2. However, statistical analysis revealed no significant difference between the two categories, except for BAPNA, where a notable increase in activity against red complex bacteria was significantly observed with tulsi as LAM.

Our study has some limitations due to its small sample size and short duration. However, this study paves the way for additional investigations into herbal treatments for periodontitis.

## Conclusions

All of the treatment approaches work to reduce periodontal pockets and enhance baseline clinical and microbiological markers. The significant improvement in mean plaque scores at the locations where curcumin gel LAM was applied suggests that curcumin has a more effective impact on plaque control compared to SRP alone. The fact that tulsi extract as LAM significantly improved BAPNA assay results and mSBI scores indicates that tulsi extract is beneficial in reducing the count of red complex bacteria and subsequent periodontal pocket bleeding. Both curcumin gel and tulsi extract have comparable clinical and microbiological control capabilities.
